# Gender differences in the transmission of risk for antisocial behavior problems across generations

**DOI:** 10.1371/journal.pone.0177288

**Published:** 2017-05-15

**Authors:** Pin Li, Jill B. Becker, Mary M. Heitzeg, Michele L. McClellan, Beth Glover Reed, Robert A. Zucker

**Affiliations:** 1 School of Public Health, University of Michigan, Ann Arbor, Michigan, United States of America; 2 Department of Psychology and the Molecular & Behavioral Neuroscience Institute, University of Michigan, Ann Arbor, Michigan, United States of America; 3 Department of Psychiatry, University of Michigan, Ann Arbor, Michigan, United States of America; 4 Department of History and the Residential College, University of Michigan, Ann Arbor, Michigan, United States of America; 5 School of Social Work and the Department of Women’s Studies, University of Michigan, Ann Arbor, Michigan, United States of America; Harvard Medical School, UNITED STATES

## Abstract

Previous studies have shown that children of alcohol use disorder (AUD) parents are more likely to develop alcohol problems as well as antisocial and other behavior problems. The purpose of this study was to examine gender discordance in the effect of early maternal and paternal influences on antisocial behaviors of boys and girls, as well as the environmental factors that moderate the parental effects. Specifically, we examined the effects of childhood and adulthood antisocial behavior of the parents on offspring antisocial behavior as young adults. We also examined whether mothers’ and fathers’ drinking problems when offspring were young children (6–8 years) affected offspring antisocial behavior as young adults (18–21 years). We evaluated 655 children from 339 families in the Michigan Longitudinal Study (MLS), a prospective study of AUD and non-AUD families. Path models were constructed in order to test for the parental contributions to offspring outcomes. We found that both mothers’ and fathers’ antisocial behavior contributed to the children’s young adult antisocial behavior. Only mothers’ drinking problems while their children were little had a significant effect on their sons’ later drinking, but not on their daughters’. These different parental effects suggest that maternal and paternal influences may be mediated by different mechanisms.

## Introduction

As the most common substance of abuse in the world, alcohol contributes not only to alcohol use disorder (AUD) but also to a host of related problems such as violence, physical abuse, traffic accidents, fetal alcohol syndrome and other negative outcomes as well as medical conditions and premature mortality. Problem drinking is also recognized as one type of impulsive behavior. According to the National Longitudinal Alcohol Epidemiologic Survey in 2014, approximately 16.3 million (6.8%) of U.S. adults were classified with a lifetime diagnosis of DSM-IV alcohol abuse or dependence, including 10.6 million men and 5.7 million women [1]. In addition, nearly 43% of US children were members of households with at least one adult who had abused or was dependent on alcohol. Among these children, 51.1% were male.

Children of alcoholics (COA) have been found to have heightened risk for antisocial, aggressive, and impulsive behavior [2]. A number of studies have investigated the causes of antisocial behavior (ASB) in both males and females, and both genetic and environmental influences have been described [3,4]. Prior twin studies have suggested that genetic and environmental factors that contribute to the ASB of adults are the same for males and females [5,6]. On the other hand, genetic influences may cause greater gender differences in ASB during adolescence when girls go through puberty earlier than boys [7]. Hicks et al found that there was an increasing genetic variation and heritability of ASB for men as they grow older, but a decreasing heritability for women with the increase of age [8]. Similarly, alcohol consumption from early adolescence to young adulthood appears to be influenced by genetic factors and shared environmental factors, and the environmental effect is gender-specific [3,9]. Prior studies have not evaluated the role of the gender of parent as well as the child in shaping these effects.

The gender of a child plays a role in the risk for developing alcohol use disorder (AUD) and other negative consequences faced by COAs. We should note that we use the term ‘gender’ throughout this article to refer to the combined effect of biological and sociocultural factors that contribute to differences between males and females. While both male and female COAs are more likely to develop AUD than children with non-AUD parents, the risk is even higher for male COAs [10]. In addition, male COAs with antisocial parents are more susceptible to intellectual, cognitive, and academic deficits than non-COA peers [11]. At young ages, COAs also have more externalizing behaviors such as aggression and violence, as well as internalizing behavior such as depression and anxiety, as reported by their parents [12]. Boys show more behavior problems than girls in both domains, and older children have more internalizing problems than younger children [10]. However, there is huge variability in the development of problems in COAs, which indicates there are multiple risk factors involved.

As shown by study of monozygotic (MZ) and dizygotic (DZ) twins, addictions are moderately to highly heritable, with a heritability of 50% for AUD and 60–70% in cocaine and opiate addiction [13]. The familial transmission of alcohol and drug dependence implies that genetic factors could affect individual risk for alcoholism [14]. For example, the GABA receptor gene *GABRA2* has been associated with adult alcohol dependence as well as with externalizing behaviors such as impulsiveness [15], likely due to an influence on incentive motivation neurocircuitry in adolescence [16].

In addition to genetic factors, environmental influences on COA development are also important and complex. The adult problems of COAs can, at least in part, be traced back to their experiences in infancy and early childhood. A family’s low socioeconomic status (SES), and high family psychopathology (e.g., low cohesion, high conflict, and poor problem-solving skills) can increase the COA’s risk of behavior disorders as well as of AUD [17]. Neighborhood residential instability in childhood and other environmental changes from early childhood to adolescence also contribute to the development of late adolescent AUD [18]. On the other hand, COAs with more sources of support in their childhood and youth cope more effectively with the trauma of growing up in an AUD family, which indicates the protective effect of caregiving from relatives and community [19,20]. Identifying environmental factors that moderate the genetic effects on the development of COAs is therefore of great importance.

Importantly, it has been demonstrated that ethanol can cause epigenetic effects (that is, changes in how genes are expressed without changing the underlying DNA). Specifically, site selective acetylation, methylation, and phosphorylation of histones due to alcohol consumption affect the expression of genes and can be transmitted across generations [21]. A recent animal study showed that the Pomc gene, which controls neuroendocrine-immune functions, can be imprinted by fetal alcohol exposure and hypermethylated through three generations. Additionally, the alcohol epigenetic marks on the Pomc gene are maintained only in the males, which indicates a gender difference in epigenetic transmission [22].

Parents’ AUD may contribute substantially to the behavior of their children through a series of complex biological, psychological, and social mechanisms, and each of these mechanisms is likely to have an effect on gender differences, although such differences have not been well studied. The effects of parental AUD on children may be direct, as conveyed by genetic or epigenetic influence, and also indirect, by way of the negative behaviors they show towards their children, including less attention yet more physical punishment and abuse. An association has been reported among COAs between experiencing maltreatment and cumulative stressful events in early life, and the early onset of both problem drinking in adolescence as well as alcohol and drug dependence in early adulthood. These outcomes could be the result of gene expression changes in the mesolimbic dopamine reward pathway, and thus would be epigenetic [23]. The AUD of one parent may also affect his/her relationship with the spouse, whose behavior also influences the children. For example, the drinking problems of the father may contribute to the externalizing/internalizing behaviors of the wife [24], which is a mediation effect on the behavior of children. One parent’s AUD may also moderate the influence of the spouse on the children. The cohesion and conflict in a family thus may influence the strength of the relationship between parents and children and relate to their antisocial and delinquent behaviors [25]; in fact, COAs were found to be more emotionally reactive to marital conflict than non-COAs [26]. However, the role of gender is less clear on these additive effects.

Current research tends to mainly focus on the behavior of boys in families with AUD, especially fathers with AUD, since AUD in males is more prevalent. However, the problem of addiction and drug use in women is increasing and maternal influences on children are also important [27]. Compared with paternal variables, maternal variables were even stronger predictors of three-year-old sons’ problem behaviors [27]. The development of girls is of equal importance to boys; however, their different performance in response to paternal and maternal influence has been less frequently studied.

In the analyses presented here we examined the prediction that the ASB of parents, during their childhood and as the parents of young children would have long-term effects on the ASB of their children as young adults. We included in our model problem drinking behavior for the father and the mother that was present when the children were 6–8 years old. We investigated gender differences in how boys and girls responded to parental behavior. We hypothesized that the fathers’ problem drinking behavior would have greater effect on the children’s ASB as young adults than the problem drinking behavior of mothers. We also predicted that the ASB of the father would have a greater effect on boys than on girls. Finally, since family cohesion can differentially mediate the effects of parental drinking and ASB on boys and girls, we included parental perception of family cohesion on boys’ and girls’ ASB in this model.

We report here that both mothers’ and fathers’ ASB had effect on children’s ASB as young adults. Compared to fathers, the mothers’ drinking behavior early during a child’s life was a stronger predictor of boys’ ASB but not of girls.

## Methods

### Sample and procedures

This study is part of the ongoing Michigan Longitudinal Study (MLS) of families with or without AUD, which started in the 1980’s [28,29]. AUD families were categorized as such via the drinking behavior of the adult men in the family; all men with drunk-driving convictions in a four-county area with a blood alcohol concentration of at least .15% (if first conviction) or at least .12% (if a previous drinking-related legal problem had occurred) were potential candidates for study enrollment. They also needed to meet diagnosis for probable/definite AUD. The original recruitment of fathers used Feighner criteria [30] to diagnose AUD, and participants were later rediagnosed using DSM-IV alcohol-use disorder criteria as the study progressed, data were collected prior to DSM-V criteria availability [29,31]. Subjects were required to have at least one 3- to 5-year-old biological son and to be living with the child and his biological mother at the time of recruitment. The AUD status of the mothers was free to vary. Female siblings joined the project several years later (when the targeted boys were at ages 6–11 years old) when funding became available to include girls. A contrast/control group of non-AUD families (neither parent with a history of substance use) was recruited through door-to-door canvassing in the same neighborhoods as the AUD families. During the canvassing, an intermediate-risk group (AUD fathers but without a history of alcohol-related legal or drunk-driving problems occurring during the life of their child) was noticed and recruited at the same time. Of all the 471 recruited families, 45% are AUD families with drunk-driving fathers (court AUD families), 21% are AUD families from the same neighborhoods (community AUD families), and 34% are non-AUD families (control families). This research was approved by the University of Michigan Medical School Institutional Review Board (IRB). Children under 9 years old provided verbal consent (and written consent later as they grew older), and their parents provided written consent with assigned consent forms. The data for this study were analyzed anonymously.

Families received extensive assessments at baseline (Time 1 with male target child at age 3 to 5) and every 3 years thereafter (e.g., Time 2 with male target child at age 6 to 8). At each wave, data were collected from all family members by interviews, self-report questionnaires, reports by collateral Informants such as spouses, parents, and teachers, as well as children's reports of their experiences with their parents [31].

In the present study we included all children who had completed Time 6 (when they were early emerging adults at 18- to 20-years of age); this pool included 655 children from 339 families. Among the children who were old enough for Time 6 assessment when the present study was conducted (about 80% of the total children in the MLS), the retention rate of children from Time 1 was approximately 95%. The sample consisted of 47% court AUD families, 21% community AUD families and 32% non-AUD families. The percentage of families in each of the categories was consistent over the years indicating no bias in attrition. At Time 6, the mean age of fathers was 48 years old and mothers was 46 years old. Our analytical model examines the relationship of parents’ ASB at T1 with the adulthood ASB of their offspring. It also examines the effects of other factors, such as parents’ drinking problems, parents’ social support, and family cohesion and conflict at T1 and T2 on the ASB of offspring as young adults. The purpose of this approach was to determine whether there are potential long term effects of parental behavior on the adult behavior of their offspring.

### Measures

#### Antisocial behavior

The Antisocial Behavior Checklist has 45 items that ask about frequency of aggressive and antisocial activities in childhood and adulthood [32,33]. The scores for each item range from 0 to 3 (0 = never, 1 = rarely, 2 = sometimes, 3 = often), with higher scores indicating a higher frequency of the behavior. This instrument also differentiates AUD from non-AUD adults, with higher ASB scores among the AUD compared to the non-AUD males [34]. In this study, the antisocial indices in childhood and adulthood of parents at Time 1 were used to predict the total antisocial scores of children at Time 6.

#### Social support

The Norbeck Social Support Questionnaire, using an interview format, evaluates the affect, affirmation and aid components of supportive transactions [35]. The measure describes the individual’s structural support network with significant people in his or her life from several perspectives. Social support is evaluated by Length of Time Known, Feeling Alike with the other, Acceptance, Trust, Monetary Support, Emotional Support, Sickness Support, Crisis Support, Love, and Desire to Imitate, with a scale of 1 to 5 (“not at all” to “a great deal”). The total social support score for parents (calculated as the product of total number of persons in one’s support network and average level from all sources) at Time 1 was used for analysis.

#### Drinking and drug history [36]

This questionnaire incorporates items from the 1978 National Institute on Drug Abuse Survey [37], from the American Drinking Practices Survey [38] and from the V.A. Medical Center (University of California, San Diego) Research Questionnaire for Alcoholics [39]. All of the items have been extensively used in a variety of survey and clinical settings. They provide data on quantity, frequency and variability of alcohol and drug use, and troubles related to the use of these substances. The total number of drinking problems for parents at Time 2 was used to describe their alcohol abuse.

#### Family conflict and cohesion

The Family Environment Scale describes dimensions of the family climate with which each family member must cope [40]. The instrument provides scores in areas that have previously been significantly implicated in AUD and drug abusing families: e.g., Cohesion, Conflict, Moral-Religious Emphasis, and Achievement Orientation. The Family Environment Scale provides insights into the perceptions of different family members. The Cohesion subscale assesses the extent of commitment, concern, and support provided by family members to one another. The Conflict subscale assesses the extent of open aggression, anger, and conflicted interactions among family members. These two scales reported by parents at Time 1 were used to evaluate the family climate.

### Analytic approach

The 655 children were matched with their parents’ variables when they were at Time 1 (3–5 years old) and Time 2 (6–8 years old). The outcome, the ASB of the children at Time 6, is a complete variable, but there are some missing data in each of the 12 parent variables for the 339 parental pairs. Overall, there were 6.9% missing data among all variable values. We assumed the data are missing at random (MAR), which is appropriate because the variables for parents tend to be associated and other variables in the dataset can be used to predict missingness for a given variable. We adopted a multiple imputation approach to impute 10 sets of complete data by Markov Chain Monte Carlo with multiple chains using SAS PROC MI. This procedure is designed for continuous variables with continuous covariates for arbitrary missing data [41]. Then SAS PROC MIANALYZE was used to aggregate the 10 sets of mediation and moderation models to get estimated parameters and their variances.

To study the different effects of fathers and mothers on boys and girls, we used a mediation-moderation model to examine the effect of parents’ adulthood ASB on their children’s adult ASB, with the mediation effect involving parents’ drinking problems. A Linear Mixed Model was used to adjust the correlation between siblings. We also tested the moderation effect of family cohesion and conflict on parents’ adulthood ASB and the moderation effect of total social support on parents’ drinking problems. To compare the difference between risk groups, we also built models for non-AUD (control) families and AUD (court AUD and community AUD) families separately. We tested the effect of parents’ childhood ASB on their own adulthood ASB, and on their offsprings’ adult ASB, which involves a longitudinal influence of approximately three decades.

## Results

### Descriptive statistics

Table 1 lists descriptive statistics for the 339 pairs of parents. All of the variables were significantly positively correlated between father and mother (p<0.01), and the correlation coefficients ranged from 0.225 to 0.478. By paired comparison, fathers had significantly higher childhood ASB, adult ASB, and number of drinking problems than mothers, while mothers had significantly more social support than fathers (p<0.01). Regarding the evaluation of family climate, fathers and mothers had similar opinions about family cohesion, but mothers reported significantly more family conflict than fathers (p<0.01). S1 and S2 Tables lists the descriptive statistics for AUD families and non-AUD families respectively. The childhood ASB and adult ASB as well as number of drinking problems for both parents were higher in AUD families compared with non-AUD families (S3 Table). The differences between fathers and mothers were also larger in AUD families. The correlations were similar in AUD and non-AUD families.

**Table 1:** Paired comparison of parents variables: Mean, standard deviations (SDs), differences, and correlation.

Variable	Parent	Mean (SD)	Min.	Max.	Differences	Correlation
Childhood Antisocial Behavior	Mother	6.43(4.78)	0	28	-3.28**	0.299**
	Father	9.71(7.34)	0	42		
Adulthood Antisocial Behavior at Time 1	Mother	4.58(3.43)	0	22	-3.77**	0.307**
	Father	8.35(6.86)	0	56		
Number of drinking problems at Time 2	Mother	0.98(2.15)	0	14.39	-2.04**	0.225**
	Father	3.02(4.93)	0	24.36		
Total social support at Time 1	Mother	28.17(13.09)	3.91	77.91	4.19**	0.360**
	Father	23.98(12.23)	3.36	69.25		
Family cohesion at Time 1	Mother	7.30(1.89)	0	9	0.07	0.361**
	Father	7.37(1.62)	0	9		
Family conflict at Time 2	Mother	3.69(2.16)	0	9	0.29**	0.478**
	Father	3.40(2.24)	0	9		

**: Significant at the 0.01 level (2-tailed)

Of the 655 children from 339 families, 199 were girls and 456 were boys. At Time 6, they were at average age 19.6 ± 2.0 years old; the mean ASB for girls was 4.04, which was significantly lower than boys (7.15), as shown in Table 2. In both non-AUD and AUD families, on average boys had significant higher ASB than girls (2.42 higher in non-AUD families and 3.52 higher in AUD families). The mean ASB for girls in AUD families was 2.27 higher than girls in non-AUD families (p<0.001), and the mean ASB for boys in AUD families was 3.37 higher than boys in non-AUD families (p<0.001).

**Table 2:** Descriptive statistics of children’s antisocial behavior at Time 6.

Family Type	Children	N	Mean (SD)	Min.	Max.	Differences
AUD families	Girls	136	4.76(4.85)	0	25	-3.52**
	Boys	303	8.28(8.60)	0	77	
Non-AUD families	Girls	63	2.49(2.58)	0	11	-2.42**
	Boys	153	4.91(4.55)	0	27	
Combined	Girls	199	4.04(4.39)	0	25	-3.11**
	Boys	456	7.15(7.65)	0	77	

**: Significant at the 0.01 level (2-tailed)

Tables 3 and 4 display the potential correlations between all variables for mother and father separately. All mothers’ variables were significantly correlated with each other except for total social support, which was only significantly positively correlated with family cohesion. The majority of the fathers’ variables were significantly correlated with each other except for total social support, which was not significantly correlated with adulthood ASB, number of drinking problems or family conflict. In general, the majority of the variables were positively correlated with each other, while total social support and family cohesion were negatively correlated with other variables. We also examined the correlations between children’s ASB and parents’ childhood ASB and adulthood ASB, and found significant positive correlations among these variables between fathers and both boys and girls, and also between mothers and both boys and girls (p<0.05, shown in S3 Table).

**Table 3:** Correlation between mothers’ variables.

Correlations:	1	2	3	4	5	6
1. Childhood Antisocial Behavior	1.00					
2. Adulthood Antisocial Behavior at Time 1	0.55**	1.00				
3. Number of drinking problems at Time 2	0.34**	0.31**	1.00			
4. Total social support at Time 1	-0.07	-0.09	-0.04	1.00		
5. Family cohesion at Time 1	-0.23**	-0.26**	-0.17**	0.17**	1.00	
6. Family conflict at Time 2	0.17**	0.24**	0.19**	-0.03	-0.47**	1.00

**: Significant at the 0.01 level (2-tailed)

**Table 4:** Correlation between fathers’ variables.

Correlations:	1	2	3	4	5	6
1. Childhood Antisocial Behavior	1.00					
2. Adulthood Antisocial Behavior at Time 1	0.72**	1.00				
3. Number of drinking problems at Time 2	0.25**	0.42**	1.00			
4. Total social support at Time 1	-0.14*	-0.11	-0.05	1.00		
5. Family cohesion at Time 1	-0.18**	-0.25**	-0.20**	0.13*	1.00	
6. Family conflict at Time 2	0.24**	0.27**	0.15*	-0.04	-0.43**	1.00

*: Significant at the 0.05 level (2-tailed);

**: Significant at the 0.01 level (2-tailed)

### Mediation & moderation analysis

**Fig 1:**
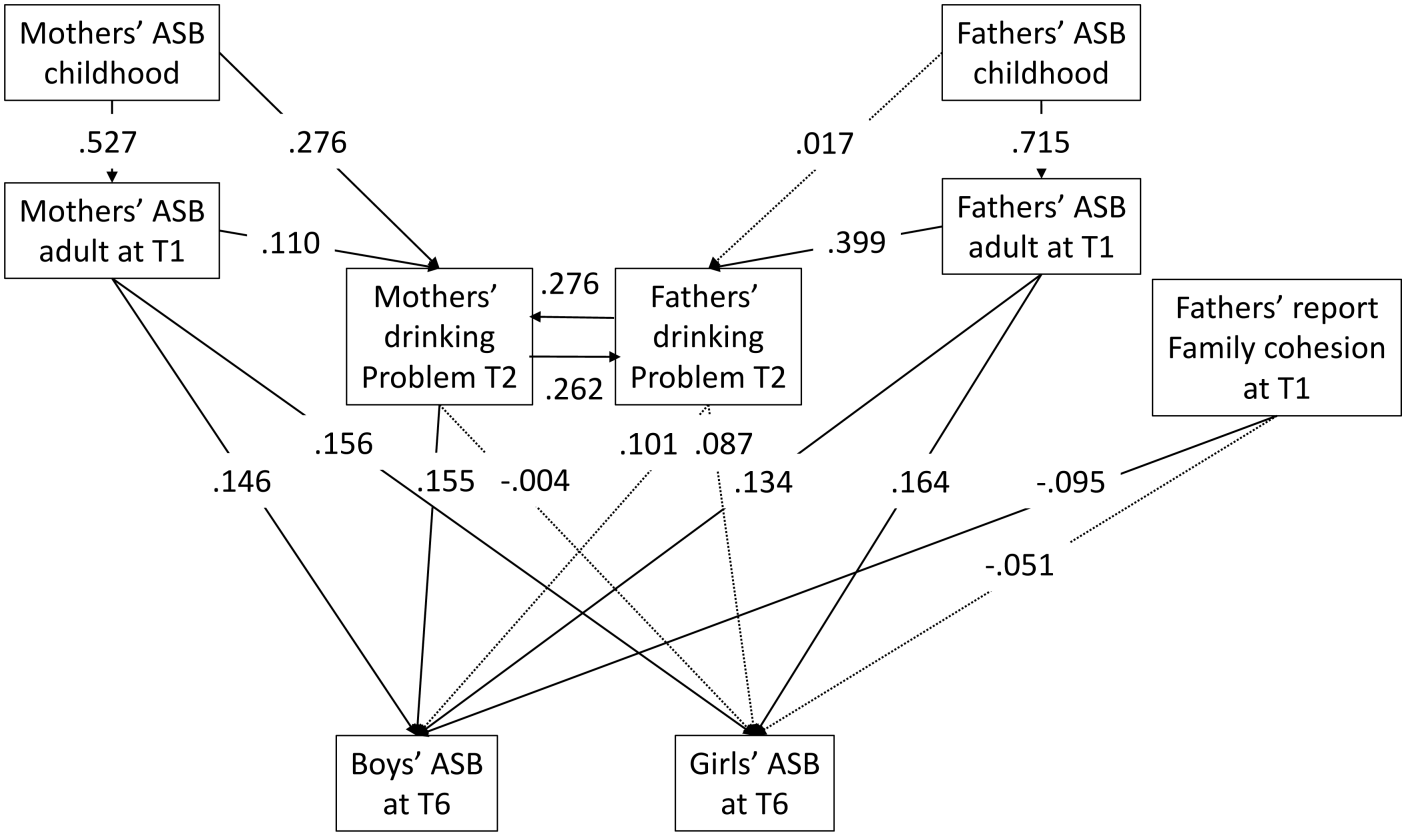
Model displaying mediation-moderation effect of parents’ antisocial behaviors and drinking problems on children’s antisocial behaviors, the effect differs by father and mother on boys and girls. The paths where standardized parameter estimates are significant at the 0.05 level are in solid lines.

As shown in Fig 1, a model of the mediation-moderation effect of parents’ ASB and drinking problems on children’s ASB was built for mothers’ and fathers’ effect on boys and girls. The standardized parameter estimate is shown on each path, which reflects the magnitude of the effect. The significant effects are shown in solid lines. Mothers’ adult ASB had significant effect on the children’s ASB (0.146 for boys, 0.156 for girls), while the effect of mothers’ drinking problems was significant only on boys (0.155). The effect of fathers’ adult ASB was significant on both boys’ and girls’ ASB (0.134 for boys and 0.164 for girls), with no direct significant effect of the fathers’ drinking problems. For the effect of family cohesion and conflict on children, only fathers’ report of family cohesion had significant negative effect on the offspring, and it was only on boys (-0.095).

For both mother and father, the effect of childhood ASB on adult ASB at Time 1 was significant, but fathers’ childhood ASB had a stronger influence on adult ASB compared with mothers’ (p<0.05) (Fig 1). The moderation effect of family cohesion and conflict on the effect of childhood ASB on adult ASB was not significant and thus not included in the model. Mothers’ childhood ASB and adulthood ASB at T1 as well as fathers’ drinking problems at T2 had an effect on mothers’ drinking problems at T2. But fathers’ childhood ASB had no direct effect on fathers’ drinking problems at T2. We can also see that fathers’ ASB at T1 had a dominant effect on fathers’ drinking problems at T2 (compared with the effect of mothers’ drinking problems, p<0.05). The moderation effect of total social support on the effect of adult ASB on drinking problems was not significant and thus excluded from the model.

These results provided mixed support for our hypotheses. We found fathers’ and mothers’ ASB both had an effect on boys and girls. Contrary to our hypothesis, only the mothers’ drinking problems had significant effect on boys, but fathers’ did not, and neither parents’ drinking had an effect on girls. The fathers’ perception of family cohesion mediated the effect on boys’ ASB.

**Fig 2:**
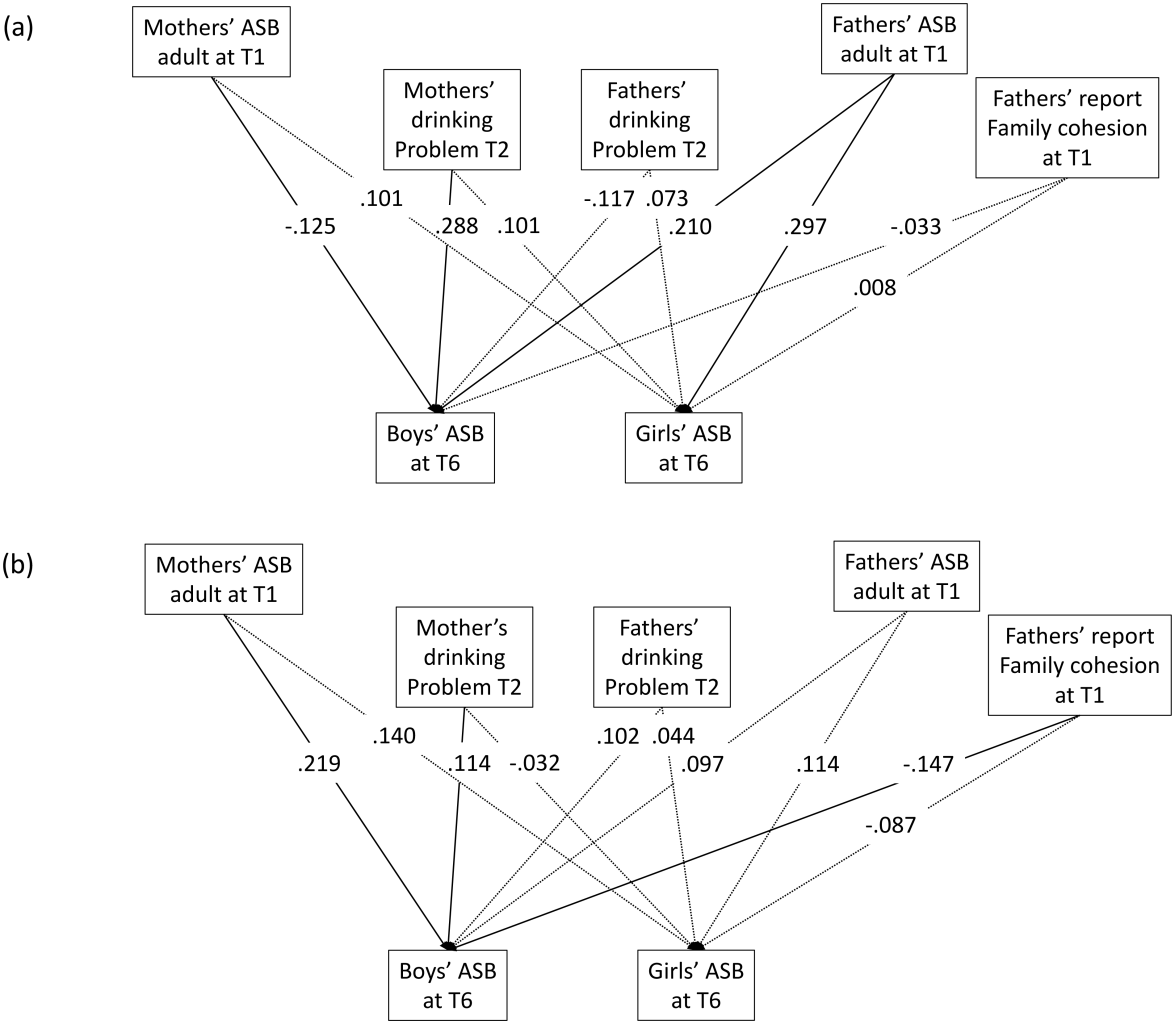
Model displaying mediation-moderation effect of parents’ antisocial behaviors and drinking problems on children’s antisocial behaviors for each risk group: (a) control group, (b)court AUD and community AUD group. The paths where standardized parameter estimates are significant at the 0.1 level are in solid lines.

Fig 2 shows the models of mediation-moderation effect of parents’ ASB and drinking problems on children’s ASB for different subject groups. For the 216 children from control families, mothers’ adult ASB and drinking problems as well as fathers’ adult ASB had significant effect on boys, and only fathers’ adult ASB was significant on girls. For the 428 children from court AUD and community AUD families, mothers’ adult ASB and drinking problems as well as fathers’ report of family cohesion had significant effect on boys, while on girls, nothing was significant. In control families, the effect of mothers’ adult ASB on boys was negative (-0.125) and the effect of mothers’ drinking problems was dominant. In court AUD and community AUD families, the effect of mothers’ adult ASB was positive and dominant compared with mothers’ drinking problems. Thus the mechanisms of how parents’ ASB and drinking problems affected children were different in AUD and non-AUD families.

## Discussion

Our findings demonstrate a difference between maternal and paternal influences on the ASB problems of their offspring during the life stage known as “early adulthood.” Mothers’ ASB had an effect on boys’ ASB and this effect was mediated by mothers’ drinking problems. For girls, the influence was from mothers’ adult ASB only. Fathers’ ASB had an effect on both boys’ and girls’ ASB, but there was no significant effect of fathers’ drinking problems on boys or girls.

Comparing the effects of parents’ behaviors raises important questions about the social roles of mothers and fathers, as well as sex and gender differences in the development and manifestation of AUD and ASB. Our findings are consistent with previous research demonstrating the importance of paternal influence on children’s development [42], even when fathers’ ASB tended not to happen at home and overt violence toward children was low. A study of 5 year-old twins showed that for children with fathers who have a high level of AUD, the more time they lived with their father, the more conduct problems they had, suggesting that children who resided with antisocial fathers received a “double whammy” of genetic and environmental risk for behavior problems [43]. Due to the high correlation between fathers’ ASB during their childhood and adulthood, we can’t distinguish their influence individually, but together their effect can’t be ignored.

Although it was the fathers’ drinking behavior at T1 that was the reason for recruitment, we did observe mothers with problem drinking behavior. In our study, 41% of the court AUD fathers and 46% of the community AUD fathers were married with AUD mothers, and there was a moderate correlation (0.2–0.3; values see Table 1) between maternal and paternal drinking problems and ASB, which we interpret as a form of assortative mating. Empirical studies have revealed that AUD males tended to find spouses who were the daughters of AUD fathers while AUD females may have both AUD fathers and husbands, and both men and women with ASB were likely to join an ASB mate [44]. These pairings can lead to domestic violence in the home, as confirmed by the extremely high divorce rate of the AUD and ASB families in the MLS [31], and represent risk for the next generation [45].

While men and women might both demonstrate ASB and drinking problems in these families, our study shows that mothers’ drinking behavior can bring different consequences for children especially for boys, as compared to fathers’ drinking. Even though paternal drinking problems were more serious in the majority of families, maternal drinking problems were more influential on their children’s development, possibly because mothers are usually the primary care givers. In the MLS, it was revealed that maternal drinking during boys' middle childhood predicted the number of their drinking days in middle adolescence [46]. Another study on young adult offspring of AUD parents revealed that COAs with mothers with drinking problems reported more negative childhood experiences, which could be related to the fact that mothers with drinking problems were more likely to drink regularly at home [47].

Research has also found that children of AUD mothers have more social relationship problems such as distant relationships with siblings and friends, negative interaction with classmates, and isolation within their neighborhood, all of which could be secondary stressors and further damage children’s mental health [48]. In other studies, it has been shown that teenagers who report less conformity and benevolence, but more independence, are more likely to develop ASB in both males and females [49]. Furthermore, interpersonal relationships can positively or negatively affect ASB during adolescence [49]. These findings highlight that even though the present study focuses on the long term effects of parental behavior prior to and during early childhood on ASB in young adults, the social structures in which a child lives and personality factors also affect ASB.

The analysis of the gender of the offspring indicates important differences in the transmission and modeling of ASB for boys and girls. In AUD families, the effect of parental influence was much stronger on male offspring than on female offspring. These findings suggest that with respect to paternal and maternal ASB, boys have a higher heritability than girls, which could result in more ASB in boys than girls, as we observed. While this gender difference is significant, it is important to note that we found in a previous study that even at very early ages, male and female COAs were heterogeneous populations that were distinguishable by familial subtypes, in particular, between antisocial AUD and non-antisocial AUD individuals [10]. Such heterogeneity during development of male and female COAs could be the result of paternal and maternal genetic influence as well as environmental risks. In the present study, we used ASB at Time 6 as an outcome measure; other research has shown that ASB is predictable across time in both males and females, with males having slightly higher stability of ASB from childhood to adulthood [5].

Besides the paternal and maternal family history of alcohol problems and violence, family proximal influences such as family conflict/cohesion when children are young also played an important role during the development of COAs. AUD families reported significantly more family conflict and less family cohesion than control families enrolled in the study. Marital conflict and parent–child conflict each functioned as a mediator of the association between fathers’ problem drinking and children's externalizing and internalizing problems as well of the as the association between mothers’ problem drinking and children's externalizing behaviors [50,51]. Family cohesion also mediated the relationship between parental problem drinking and adolescent externalizing behaviors for boys and girls [52]. The Dunedin Longitudinal Study also revealed that family conflict increased the risk of ASB in both male and female children, with a greater effect on boys [45]. Further, parent–child conflict interacted with parental problem drinking to moderate some domains of children's adjustment, which indicated parent–child conflict was a robust vulnerability factor for internalizing problems. Several protective factors such as parental attachment and monitoring were found to attenuate the positive association of family conflict to adolescent conduct problems; however, these factors only worked for girls and served as additional risk factors for boys by exacerbating this relationship [53].

In MLS, COAs who are being raised in AUD families consistently reported greater risk for stressors (especially, negative life events) and were also more likely to experience stressors repetitively and to rate their stressors as more severe [54]. It has been reported that socioeconomic status (SES) is a more important predictor of alcohol dependence symptoms among men compared to women [55], and that families with antisocial AUD parents are usually characterized as low SES [31]. Stresses on parents have been related to their drinking problem and ASB, which would become stressors on children through a mediation effect [56]. Early research on these children suggests that for fathers, social support and stress are each independent direct predictors of child maltreatment, while for mothers, social support is an indirect predictor of child maltreatment, which moderates the effect of stress on child maltreatment [57]. Even through early life stress can result in permanent neurohormonal changes, morphological changes in the brain, and gene expression changes involved in the development of addiction, gene–environment interactions and family and peer relationships are important for resilience in the development of psychopathology [23].

In the Dunedin Longitudinal Study, researchers focused on the ASB of independent groups of males and females from age 3–21. They found that males are more vulnerable to family risk factors, neuro-cognitive deficits, difficult temperament, and peer problems, which increase the risk for ASB. Moreover, males are more exposed to these important risk factors, except for family risk factors. The Dunedin researchers suggested that gender differences in level of various risk factors account for more than half of the gender differences in ASB [45].

Moving outward from the individual level and even family unit, the wider community environment is an important factor in AUD and ASB. Previous research by our group has shown that the more alcohol problems a man has, the more likely he is going to remain in, or migrate into, a disadvantaged neighborhood [58]. More research is needed to determine whether the same is true for women or female-headed households. Moreover, longitudinal studies encompass not only developmental changes for individuals and families, but historical changes too. In the present study, the offspring were born from 1977 to 1993. During these decades, many Michigan communities underwent significant shifts with economic dislocation, job loss, and recession [59]. With the launching of laws that escalated consequences for crime and drugs in the 1970s and 1980s, Michigan’s prison population increased by 53.8%, leading to additional dislocation and financial pressures for families in the midst of economic fallout. [60]. Beginning in 1981, fiscal conservatism reduced federal aid to local governments, and some local aid programs were eliminated while community development block grants were cut [61]. Budget cuts to schools, social services, and community organizations reduced the resources available to children and families [62]. Studying these changes and their potential interactions with individual health and family cohesion will be a focus of our future research.

One of the limitations of the current study is that it is based on a sample from a subpopulation with white AUD fathers, and as a by-product of their drunk driving offenses, is also heavily seeded for high levels of ASB. When the data collection started in the 1980s, the sample families were chosen according to paternal AUD. Maternal AUD was not recruited solely because of limited sample size and difficulty in follow up with high divorce or separation rates. Due to assortative mating, maternal alcohol problems in paternal AUD families were significantly higher than in the general population [63]. In that respect the present findings are applicable to a high-risk subset of the population but their relevance to a general population sample remains to be demonstrated. Additionally, the data were initially collected on 3–5 year-old boys. Because of funding agency decisions, the project was funded to enroll the girls several years later, which resulted in fewer numbers of females and some incompleteness of their information. Another limitation is that the items in ASB Checklist were developed to measure ASB for males, and do not represent as precisely the ASB of females, which has likely led to lower ASB scores for the females. Finally, this study is limited by its focus on the effects of parental behavior ASB early during a child’s life on the offspring’s behavior as a young adult, and has not considered the contributions of intervening events and experience on the offspring ASB. We acknowledge the importance of these other factors and plan to explore the relations among early and late experiences in future studies.

We conclude that these findings highlight the difference of maternal and paternal influences on the development of ASB in children of AUD families, and demonstrate that parental effect was stronger on boys than girls. The mechanisms mediating these factors will be important to determine. For example, the observation that the fathers’ ASB in childhood was strongly correlated with his ASB at T1 may indicate that epigenetic factors could play a role in the overall outcome for the fathers’ influences on both daughters and sons. Finally, these data indicate that it is important to include gender in analyses of the risk and protective factors for ASB.

## Supporting information

S1 Table Paired comparison of parents’ variables: Mean, standard deviations (SDs), differences, and correlation for AUD families (N = 232). (DOCX)

S2 Table Paired comparison of parents’ variables: Mean, standard deviations (SDs), differences, and correlation for non-AUD families (N = 107). (DOCX)

S3 Table Correlations between children’s ASB with parents’ ASB. (DOCX)
