# Supplementary material for: Gender differences in the transmission of risk for antisocial behavior problems across generations
Source: PLoS One. 2017 May 15;12(5):e0177288. doi: 10.1371/journal.pone.0177288 (PMC5432185; doi:10.1371/journal.pone.0177288)
Supplement: S1 Table — (DOCX) [file pone.0177288.s001.docx]

S1 Table. Paired comparison of parents variables: mean, standard deviations (SDs), differences, and correlation for AUD families (N=232)

| Variable | Parent | Mean (SD) | Min. | Max. | Differences | Correlation |
| --- | --- | --- | --- | --- | --- | --- |
| Childhood Antisocial Behavior | Mother | 7.27(5.12) | 0 | 28 | -4.06** | 0.239** |
|  | Father | 11.33(7.82) | 0 | 42 |  |  |
| Adulthood Antisocial Behavior at Time 1 | Mother | 5.17(3.58) | 0 | 22 | -5.01** | 0.233** |
|  | Father | 10.17(7.35) | 1 | 56 |  |  |
| Number of drinking problems at Time 2 | Mother | 1.32(2.40) | 0 | 14.39 | -3.19** | 0.165* |
|  | Father | 4.51(5.60) | 0 | 24.36 |  |  |
| Total social support at Time 1 | Mother | 28.03(12.87) | 0 | 76.25 | 4.51** | 0.362** |
|  | Father | 23.52(12.09) | 3.36 | 69.25 |  |  |
| Family cohesion at Time 1 | Mother | 7.13(2.01) | 0 | 9 | -0.18 | 0.316** |
|  | Father | 7.31(1.61) | 1 | 9 |  |  |
| Family conflict at Time 2 | Mother | 3.89(2.21) | 0 | 9 | 0.26 | 0.487** |
|  | Father | 3.63(2.13) | 0 | 9 |  |  |

*: Significant at the 0.05 level (2-tailed); **: Significant at the 0.01 level (2-tailed)
