# Supplementary material for: Gender differences in the transmission of risk for antisocial behavior problems across generations
Source: PLoS One. 2017 May 15;12(5):e0177288. doi: 10.1371/journal.pone.0177288 (PMC5432185; doi:10.1371/journal.pone.0177288)
Supplement: S2 Table — (DOCX) [file pone.0177288.s002.docx]

S2 Table. Paired comparison of parents variables: mean, standard deviations (SDs), differences, and correlation for non-AUD families (N=107)

| Variable | Parent | Mean (SD) | Min. | Max. | Differences | Correlation |
| --- | --- | --- | --- | --- | --- | --- |
| Childhood Antisocial Behavior | Mother | 4.66(3.36) | 0 | 28 | -1.65** | 0.231* |
|  | Father | 6.31(4.70) | 0 | 42 |  |  |
| Adulthood Antisocial Behavior at Time 1 | Mother | 3.33(2.72) | 0 | 22 | -1.17** | 0.273** |
|  | Father | 4.50(3.27) | 0 | 56 |  |  |
| Number of drinking problems at Time 2 | Mother | 0.38(1.44) | 0 | 14.39 | -0.01 | 0.137 |
|  | Father | 0.39(1.04) | 0 | 24.36 |  |  |
| Total social support at Time 1 | Mother | 28.49(13.59) | 3.91 | 77.91 | 3.49* | 0.357** |
|  | Father | 24.99(12.53) | 3.36 | 69.25 |  |  |
| Family cohesion at Time 1 | Mother | 7.67(1.55) | 0 | 9 | 0.16 | 0.478** |
|  | Father | 7.51(1.63) | 0 | 9 |  |  |
| Family conflict at Time 2 | Mother | 3.27(2.00) | 0 | 9 | 0.34 | 0.434** |
|  | Father | 2.92(2.38) | 0 | 9 |  |  |

*: Significant at the 0.05 level (2-tailed); **: Significant at the 0.01 level (2-tailed)
