# Supplementary material for: Gender differences in the transmission of risk for antisocial behavior problems across generations
Source: PLoS One. 2017 May 15;12(5):e0177288. doi: 10.1371/journal.pone.0177288 (PMC5432185; doi:10.1371/journal.pone.0177288)
Supplement: S3 Table — (DOCX) [file pone.0177288.s003.docx]

S3 Table. Correlations between children’s ASB with parents’ ASB

| Correlations with: | N | Mothers’ Childhood ASB | Mothers’ Adulthood ASB at Time 1 | Fathers’ Childhood ASB | Fathers’ Adulthood ASB at Time 1 |
| --- | --- | --- | --- | --- | --- |
| Boys | 456 | 0.23** | 0.26** | 0.15** | 0.26** |
| Girls | 199 | 0.17* | 0.22** | 0.18* | 0.26** |
| Boys in AUD families | 303 | 0.21** | 0.27** | 0.09 | 0.21** |
| Girls in AUD families | 136 | 0.11 | 0.15 | 0.07 | 0.16 |
| Boys in non-AUD families | 153 | 0.09 | -0.04 | 0.13 | 0.14 |
| Girls in non-AUD families | 63 | 0.11 | 0.25* | 0.24 | 0.30* |

*: Significant at the 0.05 level (2-tailed); **: Significant at the 0.01 level (2-tailed)
